# Comparison of Prevalence of Synovitis by Ultrasound Assessment in Subjects Exposed or Not to Self-Reported Physical Overexertion: The Monday's Synovitis

**DOI:** 10.1155/2014/563981

**Published:** 2014-11-06

**Authors:** C. A. Guillén Astete, A. Boteanu, A. Zea Mendoza

**Affiliations:** Rheumatology Department, University Hospital Ramón y Cajal, Carretera de Colmenar Viejo, Km 9,1, 28034 Madrid, Spain

## Abstract

*Objective*. To compare the proportion of synovitis detected by ultrasonographic study (USS) of the hands, in subjects with no rheumatologic known disease according to self-reported level of overexertion performed the day before.* Methods*. 407 consecutive volunteers were enrolled in a twelve-month period and underwent an ultrasound assessment of the hand. All studies were performed on Monday or Friday. Subjects were grouped according to their self-reported overexertion carried out the day before. Presence or absence of ultrasonographic findings compatible with synovitis was compared between groups.* Results*. 95.8% of those tested on Friday had made no overexertion the day before the study, while 30.2% of those assessed on Monday declared to have carried out an overexertion. Presence of carpal synovial hypertrophy, synovial fluid/effusion, and power-Doppler signal was statistically higher in subjects who carried out an overexertion the day before the study than the rest of subjects when the dominant hand was assessed. Globally, presence of any synovitis ultrasonographic finding was statistically higher in subjects who were studied on Monday than Friday (34.9% versus 12.1%) and in subjects who self-reported an overexertion the day before compared to the rest of subjects (47.7 versus 11.5%).* Conclusions*. In general, we recommend performing the USS as many days as possible after the most recent overexertion.

## 1. Introduction

Two elementary findings are associated with the definition of synovitis: synovial hypertrophy (SH) and synovial fluid/effusion (SF) [1]. Ultrasound study (USS) has been shown to be superior to clinical examination in detecting and evaluating both SH and SF [2, 3] and its performance has been recently introduced as part of many indexes designed to define crucial status of patients with rheumatic inflammatory diseases such as degree of activity, remission or to predict flares [4–6]. SH and SF are evaluated primarily by gray-scale ultrasound (GSUS) while Color Doppler (CD) and Power Doppler study (PD) are used to demonstrate activity related to SH by visualizing the microvascular blood flow in synovial and entheseal inflammation [7, 8].

Under a clinical point of view, ultrasonographic synovitis corresponds to arthritis. By the other hand, it is acceptable that there is a proportion of subclinical synovitis to be unnoticed by conventional physical examination. In fact, this capability of the USS is the main reason to consider it as part of the assessment of patients that gather remission criteria at least in clinical trials of different autoimmune inflammatory diseases and less extended in daily office practice [5–7, 9–11].

It must be taken into account that the definition of synovitis is not only related to immunologic rheumatic diseases. In fact, SF and even some grade of SH can be seen in healthy subjects or patients with osteoarthritis and its prevalence in general population has been reported from 5 to 23% [12–15].

Overexertion or traumatic injuries are potential causes of this cause of synovitis [16, 17] that can be nominated as nonautoimmune synovitis (NAS).

There is no reason to think that patients diagnosed with any autoimmune inflammatory disease cannot develop a NAS as well as an inflammatory flare. Moreover, when applying ultrasound-related indexes to assess their evolution the proportion of NAS able to be noticed will increase, because subclinical synovitis will be more probably detected. Considering that USS can not distinguish NAS from slightly autoimmune inflammatory synovitis, the study should be performed under conditions that make NAS as less as probable as possible.

In order to perform a USS able to provide useful information for clinical decision making, we must document all potential causes of NAS and take them into consideration when making the ultrasound report. Traumatic injuries are almost always easy to report by patients while overexertion is certainly more difficult to assess. The definition of overexertion is quite subjective: “excessive exertion; so much exertion that discomfort or injury may result” [18]. This definition makes it difficult to homogenize an exposure to an experimental exertion due to the fact that many other factors such as muscle mass, training, or constitution can influence the self-reported overexertion. It seems to be easier to predict when it is more probable to be exposed to an overexertion: in our experience patients disclose that on weekends they perform some activities that they are not used to do on other days.

The aim of the present study is to determine in what extent overexertion can be identified as a factor associated to NAS.

## 2. Methods

We conducted a prospective study of exposed and not exposed cases (proportion 1 : 2). An exposed case was that who carried out physical overexertion so considered by the subject himself, a day before the GSUS and PDU study. Not exposed cases (controls) were everyone else. Prior data indicate that the synovitis among healthy people has a prevalence of 5–27% [12, 13]. Assuming the true relative risk to find synovitis in control subjects relative to exposed is 0.24, we calculated a sample size of 182 exposed and 182 control subjects to be able to reject the null hypothesis that this relative risk equals 1 with probability (power) 0.8. The type I error probability was considered 0.05. We have used a continuity-corrected chi-squared statistic or Fisher's exact test and Student's *t*-test to evaluate the null hypothesis where appropriate.

This prospective study received the approval of our local ethics committee. Four hundred and seven consecutive adult subjects were enrolled from July 2011 to July 2012. Subjects were healthy volunteers or patients who consulted to the low priority unit of our A&E department due to a complaint not related to the musculoskeletal system or their relatives. The enrolment was based on order of arrival until achieving the sample size. Exclusion criteria were diagnosis of any inflammatory rheumatic disease and use of NSAID or any traumatic injury in carpal joint or hands in the last week. Ultrasound studies were performed on Mondays and Fridays, mornings only. Epidemiological data were collected and questionnaires about recent physical activity were performed before the ultrasound study (see Annex  1 in Supplementary Material available online at http://dx.doi.org/10.1155/2014/563981). Physical overexertion was considered as a self-assessment of physical activity 50% more intense than usual (from Monday to Friday) while regular activity was considered as intensity more than 0 and less than 50% than usual. Self-reported physical exertion carried out the day before the study was the grouping ordinal variable that took three values: less than regular exertion, regular exertion, and overexertion. Less and regular self-reported physical exertion was finally considered as no overexertion when comparisons were made.

A single rheumatologist performed GSUS and PDU studies. A second rheumatologist analyzed the GSUS and PDU images without knowing any data from subjects. First and second rheumatologists were fully trained in musculoskeletal ultrasonography and its field of work included not less than a hundred musculoskeletal ultrasonographies performed per month. Prior to start studies, a concordance test with 45 subjects was performed. Kappa index between both rheumatologists was 0.91 when gray scale concordance was assessed and 0.94 when Doppler signal was assessed.

GSUS and Power Doppler ultrasound PD assessment were performed in both hands using a GE Logic 9 ultrasonographer. Settings for gray scale study were Frec 13 MHz, gain 60, dynamic scale 81, and deep 2.0 cm (wrist), 1.5 (fingers). Settings for PD study were Frec 13 MHz (PD 8.0 MHz), gain 11, PRF 11, and deep 2.0 cm (wrist), 1.5 (fingers).

Joints studied were the second and third metacarpophalangeal (MCP) and carpal joints of both hands. MCP study was performed over the dorsal aspect of the second and third fingers and carpal study was performed with the probe over the Lister's tubercle and third finger position which has been recently probed as the most reliable position to get images from the carpal joint [19].

## 3. Results

A total of 2442 USS were performed over a twelve-month period on 407 consecutive subjects. Two hundred forty volunteer healthy people (workers of our hospital, people accompanying patients, medical students, and residents) and 167 patients with no complaint related to the joints studied were enrolled. Average age was 49.04 ± 18.62 (17–91) years old, with a weight of 72.51 ± 11.72 (40–114) Kg and BMI of 26.71 ± 4.89 Kg/m^2^. Female gender proportion was 52.8%. A 54.5% of USS were performed on Friday.

According to the grouping variable, the age, gender, BMI, day of study performance and proportion of right handed people were comparable (Table 1). All those characteristics were also comparable when grouping in two groups: self-reported overexertion or not (data not shown).

Level of self-reported overexertion performed the day before the USS showed a statistically significant association with the day of the week. 95.8% of those tested on Friday had made no exertion the day before the study, while 30.2% of those assessed on Monday declared to have carried out an overexertion (*P* < 0.001, contingency coefficient 0.331).

Presence of carpal SH, SF, and PD signal was statistically higher in subjects who carried out an overexertion the day before the study than the rest of subjects when the dominant hand was assessed. The proportions of those findings were comparable when the not dominant hand was assessed (Table 2). Grouping subjects by the day when the USS was performed, results were quite similar: 24.4% of subjects with HS were assessed on Friday while 75.6% on Monday and 56.0% of subjects without HS were assessed on Friday while 44.0% on Monday (*P* < 0.001); 23.4% of subjects with SF were assessed on Friday while 76.6% on Monday and 58.3% of subjects without SF were assessed on Friday while 41.7% on Monday (*P* < 0.001).

Presence of MCP joint synovitis was statistically higher in subjects who had carried out an overexertion the day before the study, both in the second and third fingers, when the dominant hand was assessed. In the not dominant hand a higher proportion of synovitis was found only in the second MCP joint (Table 3).

Considering SH, SF or PD as elementary ultrasonographic findings related to synovitis we constructed a crosstab of synovitis (any of the three findings) and the day when the study was carried out or the self-reported exertion performed the day before the USS (Table 4). Proportion of presence of any finding suggestive of synovitis was statistically higher on subjects who were studied on Monday than Friday (34.9% versus 12.1%) and on subjects who self-reported an overexertion the day before the study compared to the rest (47.7% versus 11.5%), *P* < 0.001 in both comparisons.

Presence of any synovitis ultrasonographic finding was statistically higher on subjects who were studied on Monday than Friday (34.9% versus 12.1%) and on subjects who self-reported an overexertion the day before compared to the rest of subjects (47.7 versus 11.5%), *P* < 0.001 in both cases.

Finally, a comparison between existence of any findings related to synovitis was performed among both genders and five quintiles of age. Neither age nor sex was associated with the detection of synovitis in our study.

## 4. Discussion

Under normal physiological conditions the synovial space is one of the most heavily pressured areas in the body. The synovial cavity is exposed to a high degree of mechanical stress under both normal and pathological conditions [16]. Besides mechanical stress due to the load of body weight or the performance of any unusual exertion, which affects predominantly the joint cartilage, mechanical stress following shear forces is also present. In fact, the motion of the synovial fluid during exercise induces shear forces whose biophysics has been studied in detail [17, 20]. Because cells can transduce mechanical stress into biochemical signals, numerous cellular functions can be influenced by the presence of mechanical stress and finally lead to an inflammatory condition.

Present study is, as far as our knowledge goes, the first aimed to value the impact of the day when an ultrasonographic study is going to be carried out. Under an exclusively clinical point of view, the detection of mild synovitis in a USS on a healthy subject is not a remarkable finding since it will not conduce to any particular clinical decision. However, NAS is possible in healthy subjects as well as patients with any inflammatory autoimmune disease. This is really important when the USS is planned to discard a flare in a patient that is considered, for instance, to be in clinical remission or when USS is performed under a scientific protocol to assess the impact of certain treatments.

Our study discloses that after an overexertion—considered as such by the subjects themselves—the probability of detection of synovitis is significantly higher compared to people who had carried out regular activities. In our experience and according to our results this phenomenon happens on weekends so the worst day to practice an USS is Monday. Probably the term “Monday synovitis” is not appropriate for those whose daily work is predominantly physical. In general, we recommend performing the USS as far as possible, the most recent overexertion.

Hand dominance is another aspect of interest. According to our results, the impact of overexertion is not significant on the carpal of the nondominant hand. This fact can be useful to decide what hand to be studied when it is possible to choose, but our data do not support this opportunity when studying MCP joints.

## 5. Limitations

One of the most important limitations of our study is that there is no “before-after” comparison. Our goal was mainly exploratory, using subjects without any known rheumatic condition. A “before-after” study when the date of exploration is a main variable has two biases difficult to solve: to hide when the exploration has been performed and to hide if the subject had developed an overexertion the day before. A second limitation is related to the absence of grading synovitis and the Doppler signal intensity. In patients with inflammatory conditions this assessment is quite important due to its implications in clinical decisions. In healthy subjects this issue has not been analyzed yet, as far as we know, and due of that there is no data about its clinical relevance. Finally, specific usual work conditions or fitness conditioning were variables not used to classify patients in this study. As pointed before “overexertion” is a relative definition and because of that is applicable to any subject regardless his usual activity. Assessing subjects according to their usual work conditions or fitness conditioning would require using a great amount of volunteers; however, we think that our self-assessed overexertion model simplifies this need. Further studies with a bigger sample size will allow assessing if grading ultrasonographic parameters is related to any kind of physical activity, a physical condition, or a particularly epidemiological or demographic feature.

## Supplementary Material

Questionnaire to be fulfilled by subjects before the USS. It includes the usual work conditions, history of recent exercise activities and self-assessment of recent overexertion.

## Figures and Tables

**Table 1:** Epidemiologic characteristics of the studied population according to self-reported level of exertion.

	Less than regular	Regular	Overexertion	*P*
*N*	39	240	128	
Age (mean SD)	52.56 ± 19.79	48.37 ± 17.943	49.22 ± 19.527	>0.1
BMI (mean SD)	26.11 ± 4.88	26.83 ± 5.13	26.65 ± 4.42	>0.1
Gender				
Female	22 (56.4%)	131 (54.6%)	69 (53.9%)	>0.05
Male	17 (43.6%)	109 (45.4%)	59 (46.1%)	
Day of USS				
Friday	11 (28.2%)	188 (78.3%)	16 (12.5%)	<0.05
Monday	28 (71.8%)	52 (21.7%)	112 (87.5%)	
Dominance				
Right handed	36 (92.3%)	219 (91.3%)	108 (84.4%)	>0.1
Left handed	3 (7.7%)	21 (8.8%)	20 (15.6%)	

**Table 2:** Findings in carpal joints grouped according to the hand dominance.

Findings	Dominant hand			Not dominant hand		
	No overexertion	Overexertion	*P* CC (approx. sig.)^*^	No overexertion	Overexertion	*P* CC (approx. sig.)
SH (+)	14 (34.1%)	27 (65.9%)	<0.001	6 (54.5%)	5 (45.5%)	0.333
SH (−)	265 (72.4%)	101 (27.6%)	0.277 (<0.01)	273 (68.9%)	123 (31.1%)	0.05 (NS)

SF (+)	18 (28.1%)	46 (71.9%)	<0.001	3 (50%)	3 (50%)	0.384
SF (−)	261 (76.1%)	82 (23.9%)	0.352 (<0.001)	276 (68.8%)	125 (31.2%)	0.049 (NS)

PD (+)	8 (22.2%)	28 (77.8%)	>0.001	2 (28.6%)	5 (71.4%)	0.340
PD (−)	271 (73%)	100 (27%)	0.297 (*P* < 0.01)	277 (69.3%)	123 (30.8%)	0.113 (NS)

^*^Exact sig. (2-sided) for Fisher Exact test; CC: contingency coefficient.

**Table 3:** Findings in MCP joints grouped according to the hand dominance.

Findings^†^	Dominant hand			Not dominant hand		
	No overexertion	Overexertion	*P* CC (approx. sig.)^‡^	No overexertion	Overexertion	*P* CC (approx. sig.)
2MCPSH (+)	10 (28.6%)	25 (71.4%)	<0.001	1 (12.5%)	7 (87.5%)	0.002
2MCPSH (−)	269 (72.3%)	103(27.7%)	0.255 (<0.01)	278 (69.7%)	121 (30.3%)	0.168 (<0.05)

3MCPSH (+)	10 (27.0%)	27 (73.0%)	<0.001	1 (33.3%)	2 (66.7%)	0.234
3MCPSH (−)	269 (72.7%)	101 (27.3%)	0.272 (<0.01)	278 (68.8%)	126 (31.2%)	0.065 (NS)

^†^2MCPSH: synovial hypertrophy of the second metacarpophalangeal joint; 3MCPSH: synovial hypertrophy of the third metacarpophalangeal joint.

^‡^Exact sig. (2-sided) for Fisher Exact test; CC: contingency coefficient.

**Table 4:** Global synovitis proportion among subjects grouped by day of study and self-reported exertion.

	Any synovitis related finding	No synovitis	*P* CC (approx. sig.)
Monday	67 (34.9%)	125 (65.1%)	<0.001
Friday	26 (12.1%)	189 (87.9%)	0.262 (<0.01)
Overexertion	61 (47.7%)	67 (52.3%)	<0.001
No overexertion	32 (11.5%)	247 (88.5%)	0.372 (<0.01)
