# Supplementary material for: Comparison of Prevalence of Synovitis by Ultrasound Assessment in Subjects Exposed or Not to Self-Reported Physical Overexertion: The Monday's Synovitis
Source: ScientificWorldJournal. 2014 Nov 6;2014:563981. doi: 10.1155/2014/563981 (PMC4241274; doi:10.1155/2014/563981)
Supplement: Supplementary file 1 — Questionnaire to be fulfilled by subjects before the USS. It includes the usual work conditions, history of recent exercise activities and self-assessment of recent overexertion. [file 563981.f1.pdf]

ESTUDIO SOBRE EL EFECTO DE LA ACTIVIDAD FISICA EN LA VALORACION ECOGRÁFICA DE SUJETOS SIN ENFERMEDADES REUMATOLOGICAS AUTOINMUNES

CUESTIONARIO – ACTIVIDAD FISICA

De acuerdo con los principios éticos de investigación científica que se encuentran vigentes, la información que usted aporte en este cuestionario será utilizada sola y exclusivamente para los propósitos del estudio cuyo título se señala arriba.

Fecha de realización del estudio: __ / __ / _____

Datos de filiación

Nombre ____ Fecha de nacimiento: __ / __ / _____ Sexo: _____

Mano dominante: Diestro ☐ Zurdo ☐ Ambidiestro ☐

¿Cuál es su situación laboral?

Desempleado ☐ Pensionista ☐ Trabajador activo ☐

En caso de ser trabajador activo continúe con el cuestionario. En caso contrario considere terminado el presente cuestionario.

Las siguientes preguntas se refieren a la actividad que realiza usted de forma habitual a lo largo de la semana, de lunes a viernes.

¿Qué grado de actividad física realiza usted como parte de su actividad laboral?

☐ Ninguno o casi ninguno. Mi actividad laboral es prácticamente sedentaria.

☐ Intermedia. Alterno periodos de sedentarismo con periodos de actividad física.

□ Mi trabajo es fundamentalmente físico.

¿Realiza usted deporte de forma habitual a lo largo de la semana? (Considera habitual, 2 o más veces por semana).

Si □ No □

Las siguientes preguntas se refieren a las actividades que usted realizó a lo largo del día de ayer (Desde que se levantó de la cama hasta que se acostó).

¿Realizó usted alguna actividad física distinta a las que realiza habitualmente? Esto incluye deporte que no realice habitualmente o trabajo que no realice habitualmente.

Si □ No □

La actividad física que realizó incluye el uso de las manos?

Si □ No □

En caso de haber respondido que si, ¿considera usted que la actividad que ha realizado es de mayor intensidad a la que habitualmente realiza a lo largo de la semana?

Si □ No □

En caso de haber respondido que si, ¿considera que la actividad que ha realizado usted es al menos un 50% más intensa que la que habitualmente realiza a lo largo de la semana?

Si □ No □
